# On the Tetraploid Origin of the Maize Genome

**DOI:** 10.1002/cfg.395

**Published:** 2004-04

**Authors:** Zuzana Swigonova, Jinsheng Lai, Jianxin Ma, Wusirika Ramakrishna, Victor Llaca, Jeffrey L. Bennetzen, Joachim Messing

**Affiliations:** 1 Waksman Institute of Microbiology Rutgers University 190 Frelinghuysen Road Piscataway NJ 08854-8020 USA; 2 Department of Biological Sciences and Genetics Program West Lafayette IN 47907-2072 USA; 3 Department of Genetics University of Georgia Athens GA 30602 USA; 4 Department of Biological Sciences Michigan Tech University 740 DOW 1400 Townsend Drive MI 49931 USA; 5 Analytical and Genomic Technologies Crop Genetics R & D DuPont Agriculture & Nutrition Wilmington DE 19880-0353 USA

## Abstract

Data from cytological and genetic mapping studies suggest that maize arose as a tetraploid. Two previous studies investigating the most likely mode of maize origin arrived at different conclusions. Gaut and Doebley [7] proposed a segmental allotetraploid origin of the maize genome and estimated that the two maize progenitors diverged at 20.5 million years ago (mya). In a similar study, using larger data set, Brendel and colleagues (quoted in [8]) suggested a single genome duplication at 16 mya. One of the key components of such analyses is to examine sequence divergence among strictly orthologous genes. In order to identify such genes, Lai and colleagues [10] sequenced five duplicated chromosomal regions from the maize genome and the orthologous counterparts from the sorghum genome. They also identified the orthologous regions in rice. Using positional information of genetic components, they identified 11 orthologous genes across the two duplicated regions of maize, and the sorghum and rice regions. Swigonova *et al*. [12] analyzed the 11 orthologues, and showed that all five maize chromosomal regions duplicated at the same time, supporting a tetraploid origin of maize, and that the two maize progenitors diverged from each other at about the same time as each of them diverged from sorghum, about 11.9 mya.


**Comparative and Functional Genomics**
*Comp Funct Genom* 2004; **5**: 281–284.
Published online in Wiley InterScience (www.interscience.wiley.com). **DOI:** 10.1002/cfg.395

**Conference Review**

### On the tetraploid origin of the maize genome

Zuzana Swigonova[1]*, Jinsheng Lai[1], Jianxin Ma[2#], Wusirika Ramakrishna[2##], Victor Llaca[1###], Jeffrey L. Bennetzen[2#] and Joachim Messing[1]

[1] *Waksman Institute of Microbiology, Rutgers University, 190 Frelinghuysen Road, Piscataway, NJ 08854-8020, USA*
[2] *Department of Biological Sciences and Genetics Program, West Lafayette, IN 47907-2072, USA*

**Correspondence to:*
*Zuzana Swigonova, Waksman Institute of Microbiology, Rutgers University, 190 Frelinghuysen Road, Piscataway, NJ 08854-8020, USA.*
*E-mail: zswigon@waksman.rutgers.edu*

[#] *Present address: Department of Genetics, University of Georgia, Athens, GA 30602, USA.*

[##] *Present address: Department of Biological Sciences, Michigan Tech University, 740 DOW, 1400 Townsend Drive, MI 49931, USA.*

[###] *Present address: Analytical and Genomic Technologies, Crop Genetics R & D, DuPont Agriculture & Nutrition, Wilmington, DE 19880-0353, USA.*

Received: 26 January 2004
Revised: 29 January 2004
Accepted: 3 February 2004

### Abstract

**Data from cytological and genetic mapping studies suggest that maize arose as a tetraploid. Two previous studies investigating the most likely mode of maize origin arrived at different conclusions. Gaut and Doebley [7] proposed a segmental allotetraploid origin of the maize genome and estimated that the two maize progenitors diverged at 20.5 million years ago (mya). In a similar study, using larger data set, Brendel and colleagues (quoted in [8]) suggested a single genome duplication at 16 mya. One of the key components of such analyses is to examine sequence divergence among strictly orthologous genes. In order to identify such genes, Lai and colleagues [10] sequenced five duplicated chromosomal regions from the maize genome and the orthologous counterparts from the sorghum genome. They also identified the orthologous regions in rice. Using positional information of genetic components, they identified 11 orthologous genes across the two duplicated regions of maize, and the sorghum and rice regions. Swigonova *et al.* [12] analyzed the 11 orthologues, and showed that all five maize chromosomal regions duplicated at the same time, supporting a tetraploid origin of maize, and that the two maize progenitors diverged from each other at about the same time as each of them diverged from sorghum, about 11.9 mya.** **Copyright © 2004 John Wiley & Sons, Ltd.**

**Keywords:** **maize; sorghum; tetraploidy**

It is generally believed that maize, *Zea mays* L., arose as a tetraploid. The evidence for this argument comes from the finding that the maize genome is largely (up to 72%) duplicated [1–5] and that the maize genome aligns in two chromosomal sets with other grass genomes [6], indicating the presence of two maize subgenomes. In addition, because many maize relatives from the tribe *Andropogoneae* have five haploid chromosomes in their nuclei, the most parsimonious explanation is that two maize progenitors, both bearing five haploid chromosomes, hybridized to give rise to a tetraploid maize. Despite extensive evidence supporting a tetraploid origin for maize, the exact mode of its origin remains unclear.

The primary study investigating the polyploid history of maize was by Gaut and Doebley [7], who examined the pattern of sequence divergence among 14 pairs of duplicated maize genes (homoeologues). They considered three models of tetraploid formation: autotetraploidy (intraspecific genome duplication), genomic allotetraploidy

Copyright © 2004 John Wiley & Sons, Ltd.

(interspecific genome hybridization) and segmental allotetraploidy (hybridization of two partially differentiated genomes). According to the mechanism of chromosomal pairing during cell division, they predicted a unimodal distribution of synonymous distances (*dS*) between maize duplicated genes for the autotetraploid and genomic allotetraploid models, with the single peak of the distribution referring to the time of switch from tetrasomic to disomic inheritance in the case of autotetraploidy, and to the time of divergence of the two diploid progenitor genomes in the case of genomic allotetraploidy. Bimodal distribution of *dS* was expected for the segmental allotetraploid model, with one peak referring to the divergence time of the two diploid ancestors (the more diverged part of the ancestral genomes) and another representing the time of switch from tetrasomic to disomic inheritance (onset of divergence of non-diverged part of the ancestral genomes). On the basis of a recovered bimodal distribution of *dS* between the 14 duplicated maize genes, Gaut and Doebley proposed a segmental allotetraploid origin of maize genome and estimated that the two maize progenitors diverged at ∼20.5 million years ago (mya) and that allotetraploidization occurred at ∼11.4 mya. Using sequence divergence data from two genes (*mdh* and *waxy*), they further suggested that one of the maize progenitors was a closer relative of sorghum than of the other maize progenitor.

However, 6 years later, Volker Brendel and colleagues (Iowa State University, Ames) reanalysed Gaut and Doebley's data, and extended the dataset to many additional putative duplicated maize genes. They found that *dS* follows a normal distribution, indicating a single genome duplication event estimated to occur at ∼16 mya (quoted in [8,9]). Although they used improved models for *dS* estimation (codon-based likelihood model), their conclusions are tentative because the orthology of the maize duplicates could not be effectively assessed.

In order to identify genes that are clearly related by descent (orthologous), Lai and colleagues sequenced five duplicated loci (Figure 1A) from the maize genome, *Zea mays* L. ssp. *mays* cv. B73, and the orthologous regions from sorghum, *Sorghum bicolor* (L.) Moench [10]. Their screening of the bacterial artificial chromosome (BAC) libraries, with probes for the five targeted loci of the five targeted chromosomal regions, resulted in single contiguous series of clones (contigs) for each of the regions in sorghum and in two contigs in

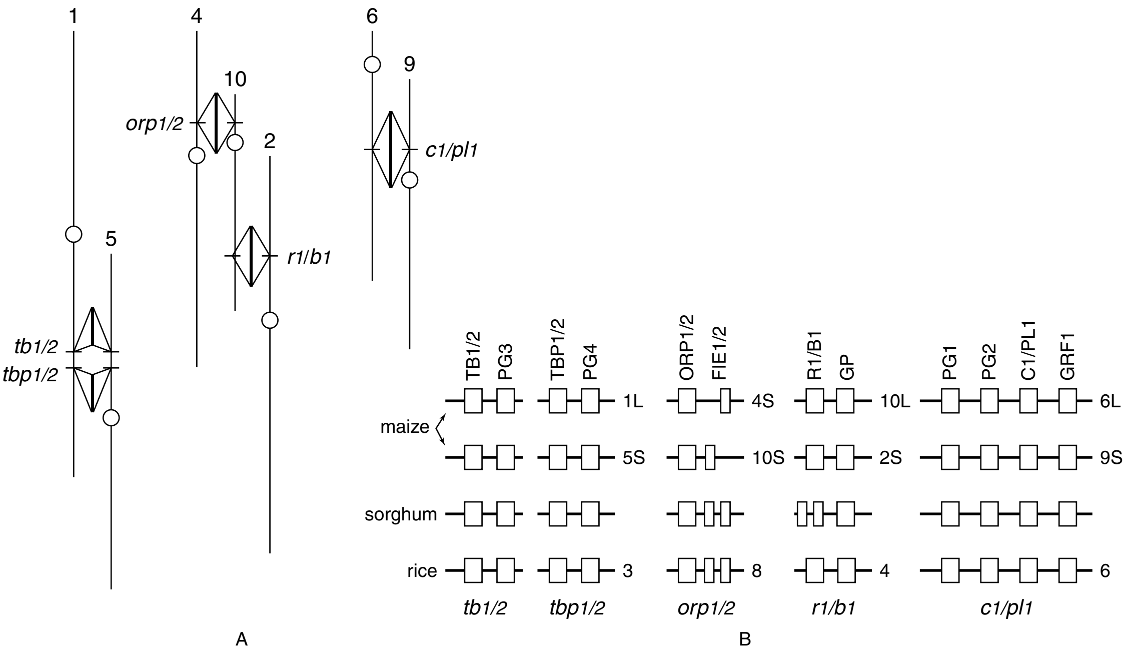

**Figure 1.** The five chromosomal segments sequenced. (A) Duplicated regions of the maize genome and their physical arrangement. (B) Orthologous genes identified within each of the five chromosomal segments. Solid lines refer to chromosomes, with circles showing the locations of centromeres. Boxes represent genes

Copyright © 2004 John Wiley & Sons, Ltd. 

each case for maize, indicating that all five regions are unique in sorghum and duplicated in maize [10]. Their finding is in agreement with the results of genetic map comparison studies of grass taxa [6,11], showing that the sorghum genome, although having the same number of chromosomes as maize (N = 10), aligns with the rice genome in a single chromosomal set.

Sequencing of 18 maize BACs and six sorghum BACs and their alignment with orthologous regions from the rice genome yielded more than four million base pairs of genetic information, within which Lai *et al.* identified 11 genes that were shared by rice and sorghum and were duplicated in homologous regions of maize (Figure 1B, [10]). Using sequence data for the 11 orthologous genes, Swigonova *et al.* reexamined the probable origin of the maize genome [12]. Having the data from three grass species, including sorghum as a sister taxon to maize and rice as an outgroup, they performed phylogenetic analyses, including maximum parsimony and maximum likelihood methods. However, the resolution within the recovered gene trees, as indicated by low bootstrap values and short internodes, was confounded by the close relationship of sorghum and maize vs. a large evolutionary distance to the outgroup, rice. Only one of the gene trees, the *r1/b1* gene tree, was found to be statistically different from a non-resolved (trichotomous) tree, showing that the *b1* locus from maize chromosome 2S is slightly closer to sorghum than it is to the *r1* locus from maize chromosome 10L, supporting an allotetraploid origin of maize. However, the internode is very short, and raises the possibility that other factors, such as different expression in different tissues, might have influenced the nucleotide substitution rates in *r1/b1* genes, leading to the present pattern of observed distances among the *r1/b1* gene orthologues. Nevertheless, the short internode in the *r1/b1* gene tree is consistent with the important finding of their study that the two maize progenitor genomes and the sorghum progenitor genome diverged from each other within a very short time period.

To examine the robustness of the results obtained from the phylogenetic analyses, Swigonova *et al.* performed analogous analyses on the 11 gene orthologues [12]. As in the two previous studies, they investigated sequence divergence among the gene duplicates of maize, but extended the examination to the sorghum gene orthologues. Using a codon-based likelihood model for distance estimation [13] and assuming that rice diverged from the ancestor of maize and sorghum at 50 mya [14], they found that rates of synonymous substitution among the 11 orthologous genes differ by 2.6-fold. In order to account for such a significant difference in rate among genes, they estimated the coalescent times for each pair of maize homoeologues and analysed the coalescent times for each pair of sorghum and maize orthologues. To calculate the coalescent time of two orthologues, they developed formulae that incorporated weighted means to account for variance information in the estimates (Lai *et al.*, unpublished). In contrast to previous studies, they used gene-specific rates of synonymous substitution to estimate the coalescent time of two orthologous sequences. Homogeneity tests [7] on coalescent times of each pair of sorghum and maize orthologues showed a common time interval. However, homogeneity tests performed on the maize duplicated genes indicated that the *tbp1/2* gene pair of maize diverged at a different time than all other gene duplicates. Moreover, *tbp1/2* also exhibited the smallest variance of synonymous distance and the smallest non-synonymous distance, despite the rather elevated rate of synonymous substitution ($11.61 \times 10^{-9}$). Therefore, they suspected that the maize *tbp1/2* gene pair is the product of a gene conversion event between the two homoeologous regions that occurred roughly 4.8 mya. All the other maize duplicated genes diverged at the same time, indicating contemporaneous duplication of the five chromosomal regions and therefore supporting whole genome duplication (tetraploidy). Since the coalescent times of each pair of sorghum and maize orthologues are statistically similar to the coalescent times estimated for each pair of maize homoeologues (except *tbp1/2*), the distance analyses furthermore support the results of phylogenetic analyses that the two progenitors of maize diverged from each other at about the same time as both of them diverged from sorghum, approximately 11.9 mya.

In conclusion, Swigonova *et al.* [12] showed that the origin of maize might be traced to a more recent time than concluded by Gaut and Doebley [7], approximately 11.9 mya when the two maize progenitors and the sorghum progenitor diverged from each other. The time of the tetraploidization cannot be determined from their data. However, if the *tbp1/2* gene pair in maize is indeed the result of

Copyright © 2004 John Wiley & Sons, Ltd. 

a homoeologous conversion, then the tetraploidization must have occurred before 4.8 mya and therefore preceded the major expansion of the maize genome by retrotransposition and gene amplification that has been estimated to occur within the last 5 million years [15].

### Acknowledgements

This work was supported by NSF Grant 9975618.

### References

1. Ahn S, Tanksley SD. 1993. Comparative linkage maps of the rice and maize genomes. *Proc Natl Acad Sci USA* **90**: 7980–7984.
2. Goodman MM, Stuber CW, Newton K, Weissinger HH. 1980. Linkage relationships of 19 enzyme loci in maize. *Genetics* **96**: 697–710.
3. Helentjaris T, Weber D, Wright S. 1988. Identification of the genomic locations of duplicate nucleotide sequences in maize by analysis of restriction fragment length polymorphism. *Genetics* **118**: 353–363.
4. McMillin DE, Scandalios JG. 1980. Duplicated cytosolic malate dehydrogenase genes in *Zea mays*. *Proc Natl Acad Sci USA* **77**: 4866–4870.
5. Wendel JF, Stuber CW, Edwards MD, Goodman MM. 1986. Duplicated chromosomal segments in *Zea mays* L.: further evidence from hexokinase isozymes. *Theor Appl Genet* **72**: 178–185.
6. Gale MD, Devos KM. 1998. Plant comparative genetics after 10years. *Genome* **282**: 656–659.
7. Gaut BS, Doebley JF. 1997. DNA sequence evidence for the segmental allotetraploid origin of maize. *Proc Natl Acad Sci USA* **94**: 6809–6814.
8. Eckardt NA. 2003. Maize genetics 2003. *Plant Cell* **15**: 1053–1055.
9. Wei G, Brendel V. 2003. The possible maize segmental allotetraploidy revisited. Plant & Animal Genome XI, poster P438: **http://www.intl-pag.org/11/abstracts/P5d_P438_XI.html**
10. Lai J, Swigonova Z, Ma J, *et al.* 2004. Frequent gene movement during grass genome evolution. Plant & Animal Genome XII, poster P337: **http://www.intl-pag.org/12/abstracts/P5a_PAG12_337.html**
11. Whitkus R, Doebley J, Lee M. 1992. Comparative genome mapping of sorghum and maize. *Genetics* **132**: 1119–1130.
12. Swigonova Z, Lai J, Ma J, *et al.* 2004. On the tetraploid origin of maize genome. Plant & Animal Genome XII, workshop W132: **http://www.intl-pag.org/12/abstracts/W29_PAG12_132.html**
13. Yang Z. 1997. PAML: a program package for phylogenetic analysis by maximum likelihood. *Comput Appl Biosci* **13**: 555–556.
14. Wolfe KH, Gouy M, Yang Y-W, Sharp PM, Li W-H. 1989. Date of the monocot–dicot divergence estimated from chloroplast DNA sequence data. *Proc Natl Acad Sci USA* **86**: 6201–6205.
15. SanMiguel P, Gaut BS, Tikhonov A, Nakajima Y, Bennetzen JL. 1998. The paleontology of intergene retrotransposons of maize. *Nat Genet* **20**: 43–45.

Copyright © 2004 John Wiley & Sons, Ltd.
